# Impact of Respiratory Syncytial Virus and Influenza Virus Infection in the Adult Population in Spain between 2012 and 2020

**DOI:** 10.3390/ijerph192214680

**Published:** 2022-11-09

**Authors:** Marco Heppe-Montero, Ruth Gil-Prieto, Jorge del Diego Salas, Valentín Hernández-Barrera, Ángel Gil-de-Miguel

**Affiliations:** 1 Department of Preventive Medicine & Public Health, Universidad Rey Juan Carlos, Avenida de Atenas s/n, 28922 Madrid, Spain; 2 Hospital Infantil Universitario Niño Jesús, Avenida Menéndez Pelayo 65, 28009 Madrid, Spain; 3 Health Promotion and Prevention, Spanish Ministry of Health, Paseo del Prado 18-20, 28014 Madrid, Spain

**Keywords:** lower respiratory tract infection, respiratory syncytial virus, influenza, hospitalization, mortality, adults

## Abstract

Respiratory syncytial virus (RSV) infection is increasingly recognized as a cause of significant morbidity and mortality in adults. We aimed to estimate the rates of age-specific hospitalization and in-hospital mortality caused by acute lower respiratory tract infections (ALRTIs) in Spain between 2012 and 2020 and to compare the relative impact of RSV and influenza virus infection in adults. We used the discharge reports from the Minimum Basic Data Set to retrospectively analyze hospital discharge data on the basis of the ICD-9-CM and ICD-10-CM diagnosis codes. A total of 1,518,244 patients were hospitalized for ALRTIs, of whom 137,794 (9.1%) were admitted for RSV-related infections and 46,288 (3.0%) for influenza-related infections. In patients aged 60 years or older, the hospitalization rates (per 100,000 population) were estimated at 1.69 (95% CI 1.68–1.70) and 2.72 (95% CI 2.71–2.73) for RSV and influenza patients, respectively. However, in-hospital mortality rates were significantly higher among RSV patients than among influenza patients, 7.91% (95% CI 7.89–7.93) (83.0% of all RSV-related deaths) versus 6.91% (95% CI 6.89–6.93) (85.6% of all influenza-related deaths), respectively (*p* = 0.007). RSV-associated in-hospital mortality increases exponentially with age, posing a greater risk for older adults, particularly frail and high-risk patients.

## 1. Introduction

Acute lower respiratory tract infections (ALRTIs), including bronchitis, bronchiolitis, influenza, and pneumonia, are a leading cause of morbidity and mortality worldwide and a public health priority [1,2]. They are caused by a mixture of viral, bacterial, and viral-bacterial coinfections [3,4,5]. Although they are most common in young children, in whom they cause up to 90% of all ALRTIs, viral infections are increasingly associated with a substantial burden of respiratory disease in adults [6,7,8]. Included among the most common viruses associated with symptomatic ALRTIs, acting either directly or as synergistic pathogens or co-factors, are respiratory syncytial virus (RSV) and influenza virus types A and B [9]. RSV infections represent a major cause of childhood morbidity and mortality worldwide in children younger than 5 years of age, especially during the first 6 months of life. In 2019, RSV infections were responsible for 33 million episodes (uncertainty range [UR] 25.4–44.6 million), 3.6 million hospital admissions (UR 2.9–4.6 million), 26,300 in-hospital deaths (15,100–49,100), and 101,400 RSV-attributable overall deaths (UR 84,500–125,200), of which more than 95% episodes and more than 97% deaths across all ages occurred in low-income and middle-income countries [10]. Although RSV infection in healthy adults has often been associated with mild disease, many studies over the last decades have raised awareness of the potential for RSV to cause severe disease in the adult population, particularly in the older patients, in patients with chronic pulmonary conditions and immunocompromised individuals, and among healthy, community-dwelling older adults [11,12,13,14]. A milestone study conducted in the US over four consecutive winters, from late 1999 through early 2003, found that RSV infection developed annually in 3% to 7% of healthy older patients (≥65 years of age), 4% to 10% of high-risk adults (those with chronic heart or lung disease), and 8% to 13% of adults hospitalized with cardiopulmonary infections. According to this study, RSV infection would account for approximately 177,525 admissions and 14,000 deaths per year [15]. In a more recent cross-sectional study evaluating excess mortality from RSV and influenza in the US from 1999 to 2018, a mean of 6549 (95% CI 6140–6958) underlying respiratory deaths were associated with RSV annually, of which 88.6% (*n* = 5800) occurred among those aged 65 years or older (14.7 [95% CI 13.8–15.5] per 100,000 population), providing evidence that RSV, apart from posing a greater risk than influenza to infants, is associated with substantial mortality among older individuals [16].

Nonetheless, while pediatricians are well aware that RSV may cause serious illness in their patients, its impact among older adults remains underrecognized by most internists [17,18]. This is partly due to the fact that the clinical symptoms of influenza-like illness are nonspecific, which can lead to misdiagnosis, particularly in individuals hospitalized during the influenza season. In addition, the low sensitivity of the diagnostic methods available in the past hampered an accurate analysis of the impact of RSV in adults [17]. Furthermore, RSV is not currently an official notifiable infectious disease monitored by the European Centre for Disease Prevention and Control (ECDC). In most European countries, including Spain, RSV surveillance is part of the influenza surveillance network, making it difficult to draw a representative picture of RSV activity in Europe [19].

Severe RSV disease and its associated costs pose a significant economic burden for the public healthcare system, which may be significantly higher in some respects than that of influenza. RSV infection is associated with a greater risk of a longer hospital stay and intensive care unit admission, particularly among older adults, in whom respiratory complications during hospitalization are more common [20,21].

At the present time, management of RSV infection in adults is mainly supportive and may include therapy with bronchodilators, supplemental oxygen, mechanical ventilation, intravenous fluids, and antipyretics [22]. Various antiviral agents and vaccines for the treatment and prevention of RSV infection are currently in clinical development, and detailed knowledge of disease epidemiology and clinical outcomes in older adults hospitalized with RSV is needed to inform policy recommendations and evaluate cost-effectiveness prior to approval [23].

The aim of this study was to estimate the age-specific hospitalization and the rates of in-hospital mortality caused by ALRTIs in the general population of Spain between 2012 and 2020 and compare the relative impact of RSV- and influenza virus-associated respiratory illnesses in older adults.

## 2. Materials and Methods

### 2.1. Study Design and Data Sources

The aim of this retrospective observational study was to collect and analyze data on hospitalizations for ALRTIs in children and adults in Spain from 2012 to 2020. We used the discharge reports from the Minimum Basic Data Set (MBDS), published annually by the Spanish Ministry of Health, to retrospectively analyze hospital discharge data containing a diagnosis of RSV, bronchitis, bronchiolitis, pneumonia, influenza, or asthma for the general Spanish population over a 9-year period, from 1 January 2012 to 31 December 2020. The MBDS reports on more than 90% of admissions to both public and private acute care hospitals in Spain and is validated for data quality and overall methodology by the Spanish Ministry of Health [24,25].

For each MBDS discharge record, we retrieved up to 9 diagnoses coded according to the International Classification of Diseases, Ninth Revision, Clinical Modification, ICD-9-CM, for cases collected between 2012 and 2015, and up to 10 diagnoses coded according to the International Classification of Diseases, Tenth Revision, Clinical Modification, ICD-10-CM, for cases collected between 2016 and 2020. For each record, the following variables were collected: diagnosis, age, and outcome (discharge/death). Eligible respiratory system diseases are listed in Table 1.

### 2.2. Data Analysis

The hospitalization rate, examined by diagnostic group, by age group and by year, was expressed per 100,000 population and 95% confidence interval (CI). The in-hospital case fatality rate, examined by diagnostic group, by age group and by year, was expressed as a percentage of deaths per inpatient population and 95% CI. Corrected population data from municipal records extracted from the National Institute of Statistics [26] were used as the denominator for the hospitalization rates. Pooled rates were calculated for the following groups of diagnostic codes: (i) Nonspecified lower respiratory tract infections, including “Acute bronchitis (codes ICD-9-CM 466.0 and ICD-10-CM J20.9)”, “Acute bronchiolitis (codes ICD-9-CM 466.1 and ICD-10-CM J21.9)”, “Bronchitis, not specified as acute or chronic (codes ICD-9-CM 490 and ICD-10-CM J40)” and “Asthmatic bronchitis (codes ICD-9-CM 493.9 and ICD-10-CM J45.9)”; (ii) RSV-related infections, including “RSV infection (codes ICD-9-CM 079.6 and ICD-10-CM B97.4)”, “Acute bronchitis due to RSV (code ICD-10-CM J20.5)”, “Acute bronchiolitis due to RSV (codes ICD-9-CM 466.11 and ICD-10-CM J21.0)” and “Pneumonia due to RSV (codes ICD-9-CM 480.1 and ICD-10-CM J12.1)”; (iii) Influenza-related infections, including “Influenza with pneumonia (codes ICD-9-CM 487.0 and ICD-10-CM J11.0)”, “Influenza with other respiratory manifestations (codes ICD-9-CM 487.1 and ICD-10-CM J11.1)”. The chi-square test was used to assess differences in proportions. Poisson regression was used to assess differences in the hospitalization rates during the study period in all the age groups. In all tests, the level of significance was set at *p* < 0.05. For the statistical analysis, we used IBM SPSS Statistics for Windows, Version 17.0 (Chicago, IL, USA) and Stata Statistical Software, Release 16 (College Station, TX, USA: StataCorp LLC).

## 3. Results

**Figure 1:**
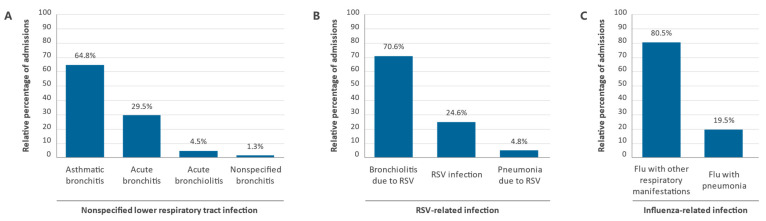
Relative percentages of admissions for the different diagnostic codes of diseases of the respiratory system in Spain between 2012 and 2020 by diagnostic group. (**A**) Nonspecified lower respiratory tract infection, (**B**) RSV-related infection, and (**C**) Influenza-related infection.

Between 2012 and 2020, a total of 1,518,244 patients were hospitalized in Spain for ALRTIs (Table 2). The median age was 65 years (range 31–81), and 55,13% of patients (*n* = 836,969) were 60 years of age or older. For all ages, the highest pooled mean annual hospitalization rate during the study period, 31.74 (95% CI 31.72–31.76) per 100,000 population, was registered among patients in the nonspecified lower respiratory tract infections diagnosis group, which included 87.9% (*n* = 1,334,162) of all patients admitted for ALRTIs during the period analyzed (Table 3). Of these patients, 864,281 (64.8%) were diagnosed with “asthmatic bronchitis”, 392,934 (29.5%) with “acute bronchitis”, 59,575 (4.5%) with “acute bronchiolitis”, and 17,372 (1.3%) with “nonspecified bronchitis” (Figure 1A).

Almost 60% of patients in this group of diagnostic codes were 60 years of age or older (*n* = 791,740; 59.3%), and their hospitalization rate was more than twice the overall rate for all ages in this group, 77.11 (95% CI 77.06–77.16) per 100,000 population (Table 3). The highest rates were observed in children under 5 years of age and older adults over 90 years. Among children under 5 years of age, the highest hospitalization rate was recorded in children younger than 1 year of age, 168.15 (95% CI 167.77–168.53) per 100,000 population (5.3-fold higher than the overall hospitalization rate for all ages); this rate fell sharply immediately thereafter until the lowest levels were reached in 5-year-olds, 9.38 (95% CI 9.35–9.41) per 100,000 population. In patients between 5 and 60 years of age, hospitalization rates showed a slightly increasing trend, from 9.18 (95% CI 9.14–9.22) to 19.45 (95% CI 19.41–19.49) per 100,000 population. From 60 years onwards, a striking, age-associated upward trend was observed in the hospitalization rate, rapidly increasing from 34.46 (95% CI 34.41–34.51) in patients aged 60–69 years to 204.03 (95% CI 203.65–204.41) in patients aged 90 years or older (Table 3).

A total of 137,794 patients were admitted for RSV-related infections during the study period, accounting for 9.1% of all patients hospitalized for ALRTIs (Table 2). Of these, 97,241 (70.6%) were diagnosed with “acute bronchiolitis due to RSV”, 33,914 (24.6%) with “RSV infection” and 6639 (4.8%) had “pneumonia due to RSV” (Figure 1B). The hospitalization rate (per 100,000 population) was estimated at 3.28 (95% CI 3.27–3.29) for all ages and for patients aged 60 years or older, who accounted for 12.6% of all RSV-related hospital admissions (*n* = 17,312), it was 1.69 (95% CI 1.68–1.70) (Table 3). Rates by age group were disproportionately higher in children under 1 year of age, reaching 263.42 (95% CI 262.97–263.87) per 100,000 population (i.e., 80-fold higher than the overall hospitalization rate for all ages). It dropped rapidly to 33.35 (95% CI 33.17–33.53) in children aged 1 year and 5.99 (95% CI 5.95–6.03) in children aged 2 to 4. From 5 years of age onwards, the hospitalization rate remained steadily below that level until a slightly increasing trend was observed from approximately 60 years onwards, reaching a relatively small 5.56 (95% CI 5.49–5.63) per 100,000 population in patients aged 90 years or older (Table 3).

**Figure 2:**
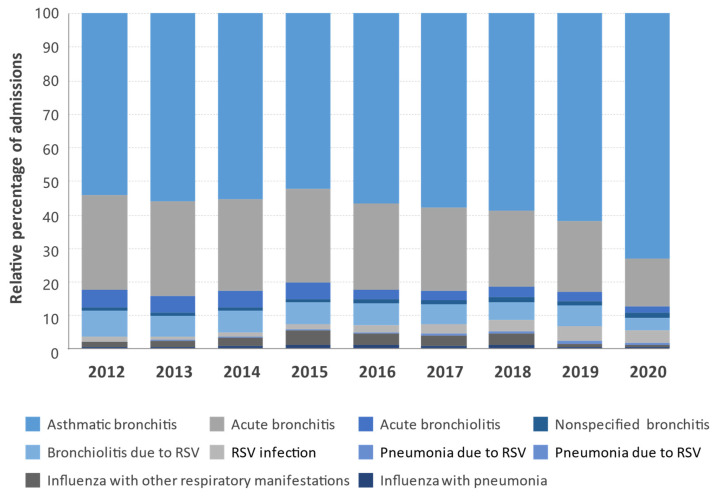
Distribution of the relative percentages of admissions for the different diagnostic codes of diseases of the respiratory system in Spain between 2012 and 2020 by year.

Only 3.0% (*n* = 46,288) of all patients admitted for ALRTIs were classified as having influenza-related infections, the lowest rates among the three diagnostic groups analyzed during the study period (Table 2). Of these patients, 37,249 (80.5%) were diagnosed with “influenza with other respiratory manifestations”, and 9039 (19.5%) had “influenza with pneumonia” (Figure 1C). The hospitalization rate (per 100,000 population) was estimated at 1.1 (95% CI 1.1–1.1) for all ages and 2.72 (95% CI 2.71–2.73) for patients aged 60 years or older. This latter group accounted for 60.3% (*n* = 27,917) of all influenza patients. Although the differences were less pronounced, the distribution of hospitalization rates across the different age groups showed a symmetrical U-shaped curve similar to that observed in patients in the nonspecified lower respiratory tract infections group, with higher rates in children under 5 years and adults over 60 years of age. The highest hospitalization rates (per 100,000 population) were registered in children under 1 year of age, 7.14 (95% CI 7.05–7.23), and older patients aged 90 years or older, 6.7 (95% CI 6.62–6.78), 6.5- and 6.1-fold higher than the overall hospitalization rate for all ages in the group (Table 3). The relative percentages of admissions for the different diagnostic codes by year of study are shown in Figure 2.

**Figure 3:**
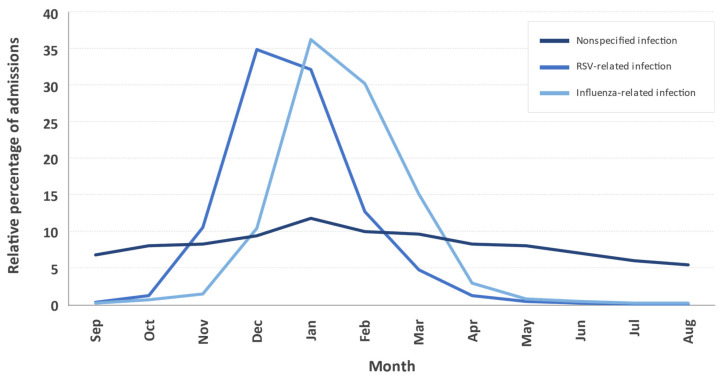
Monthly distribution of the relative mean percentage of admissions for the indicated diagnostic groups throughout the year in Spain between 2012 and 2020.

Admissions for nonspecified lower respiratory tract infections occurred in all seasons, being more frequent in the winter months. In contrast, admissions for RSV-related infections were recorded almost exclusively during the fall and winter, peaking between December (35%) and January (32%), about a month earlier than those recorded for influenza-related infections, which peaked between January (36%) and February (30%) (Figure 3).

**Figure 4:**
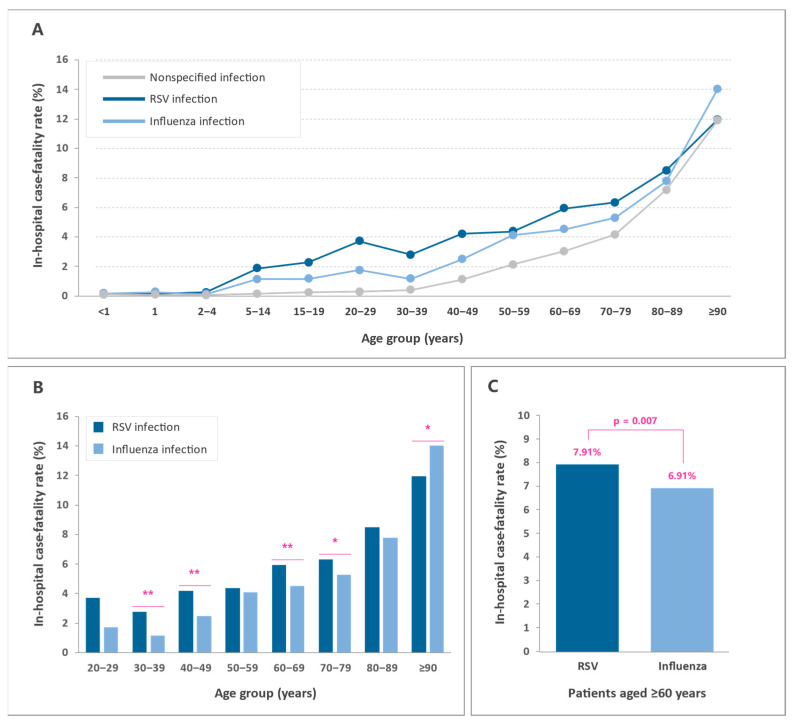
In-hospital case fatality rates for lower respiratory tract infections in Spain between 2012 and 2020. (**A**) Mortality rates for nonspecified infection, RSV infection, and influenza infection by age group. (**B**) Mortality rates for RSV infection and influenza infection in adult patients (≥20 years) by age group. (**C**) Mortality rates for RSV infection and influenza infection in patients aged 60 years or older. Rates are expressed as the percentage of deaths per inpatient population. * *p* ≤ 0.05; ** *p* ≤ 0.01.

The in-hospital case fatality rate was highest among patients admitted for influenza-related infections, 4.87% (95% CI 4.67–5.07), followed by patients admitted for nonspecified lower respiratory tract infections, 3.85% (95% CI 3.82–3.88), and patients admitted for RSV-related infections, 1.20% (95% CI 1.14–1.26) (Table 4). In all three diagnostic groups, in-hospital mortality increased exponentially with age, reaching levels about 100-fold higher in patients aged 90 years or older than in children under 1 year of age. In contrast to what was observed in patients admitted for nonspecified lower respiratory tract infections, in which the in-hospital case fatality rates remained at minimum levels until they increased exponentially from the age of 40, in patients admitted for both RSV and influenza infection, mortality increased rapidly from as early as 5 years of age (Table 5 and Figure 4A).

In the adult population (≥20 years), in-hospital mortality was higher among RSV patients than among influenza patients for all age groups analyzed, except in patients aged 90 years or older (Table 5 and Figure 4B). Among adults aged 60 years or older, the in-hospital mortality was significantly higher for RSV patients than for influenza patients, 7.91% (95% CI 7.89–7.93) versus 6.91% (95% CI 6.89–6.93), respectively (*p* = 0.007) (Figure 4C).

## 4. Discussion

In this retrospective nationwide study, we provided data on the age-specific burden of ALRTIs in the general population in Spain from 2012 to 2020 and compared the relative impact of RSV- and influenza virus-associated respiratory illnesses in older adults. During the 9-year study period, more than 1.5 million admissions due to ALRTIs were recorded in our country, of which 9% were associated with RSV infection and 3% with influenza virus infection. The distribution of hospitalization rates across the different age groups showed a symmetrical U-shaped curve in all diagnostic groups, with higher rates in children under 5 years, particularly among RSV patients, and adults over 60 years of age, in whom a steep age-dependent upward trend was observed. In patients admitted for RSV and influenza infection, the in-hospital case fatality rate, in contrast to the hospitalization rate, was very low in children under 2 years of age (<0.25%) and increased exponentially thereafter. Although the age-associated increase in the in-hospital mortality rate followed a similar pattern for both RSV and influenza infections, mortality among RSV patients was higher than among influenza patients in all age groups from 2 years onwards, except in patients aged 90 years or older. Of note, the mortality rate for RSV in adults aged 60 years or older was significantly higher than that for influenza (7.91% vs. 6.91%, *p* = 0.007). However, differences in mortality should be interpreted with caution, particularly among older adults, since a significant percentage of them had presumably been immunized against seasonal influenza. According to data from the Spanish Ministry of Health, the mean coverage of influenza vaccination in patients aged ≥65 during the study period was estimated at 57.0% (SD 4.1). Vaccination coverage remained fairly stable from the 2012–2013 to 2019–2020 seasons (mean 55.7% [SD 0.9]), increasing to 67.7% in the 2020–2021 season due to the COVID-19 pandemic.

Epidemiological and retrospective cohort studies have shown that RSV infection is a significant cause of morbidity and mortality in the adult population that is at least comparable to that of seasonal influenza A and B [11,15,17,27,28,29]. Although a retrospective study of hospitalizations for RSV among adults (≥20 years) in the US from 1997 to 2012 reported fewer admissions than for influenza, these episodes were more severe and were associated with higher overall in-hospital mortality, 6.2% versus 3.0% (ratio 2.1), greater use of mechanical ventilation (16.7% vs. 7.2%), and longer length of stay (6.0 vs. 3.6 days). The in-hospital mortality ratio between RSV and influenza patients in this retrospective study was quite similar to that found in our study [30]. In a retrospective cohort study of adult patients (≥18 years) admitted for RSV infections during 2013–2015 in the Republic of Korea, the adjusted hazard ratio (HR) for death due to RSV versus influenza was estimated at 2.32 (95% CI 1.17–4.58) [31].

There is evidence of epidemiological differences between RSV and influenza patients, including older age, higher age-adjusted Charlson comorbidity index (CCI) scores, as well as clinical presentation differences [20,32,33]. Moreover, evidence of bacterial superinfection during the disease course has been reported in about 15% of RSV patients, a factor that contributes significantly to higher mortality (HR for death, 2.02 [95% CI 1.11–3.66]) [27].

Although RSV in this study accounted for only 1.4% of all respiratory viral infections in adult patients (≥20 years), it is a potentially severe cause of respiratory hospitalization and mortality and should not be looked upon as trivial. For comparison, influenza infection, which has a disproportionate impact on the adult population and individuals with underlying high-risk medical conditions, accounted for 2.5% of all respiratory infections requiring hospitalization. However, the in-hospital case fatality rate among patients ≥ 20 years of age admitted for RSV infection was 7.3%, around 1.3-fold higher than in patients admitted for influenza infection, estimated at 5.8%. Our results are in line with those reported in other studies assessing the impact of RSV and influenza infection in hospitalized adult patients. In a retrospective cohort study of adults (≥18 years) admitted to four Canadian hospitals with laboratory-confirmed RSV infection between September 2012 and June 2013 (*n* = 86), the in-hospital mortality rate was estimated at 6% [28]. Furthermore, in a retrospective study evaluating the morbidity and mortality in adult patients (≥18 years) hospitalized between October 2017 and April 2018 with RSV infection (*n* = 113) or laboratory-confirmed influenza A/B (*n* = 526) in Israel, no statistically significant differences were found in the 30-day or 90-day all-cause mortality rates between the two diagnoses (5% vs. 7% [*p* = 0.4] and 11% vs. 11% [*p* = 0.9], respectively). Compared with patients with influenza, patients with RSV infections in this study were similar in age but had high age-adjusted CCI scores and more frequently had major systemic comorbidities [29]. Similarly, in another retrospective cohort study conducted in adults (≥18 years) admitted to three acute care general hospitals in Hong Kong with virologically confirmed RSV infection (*n* = 607) or seasonal influenza (*n* = 547) between 2009 and 2011, the crude 30-day all-cause mortality rates for patients with RSV and influenza were comparable, 9.1% versus 8.0%, respectively (*p* = 0.538). The 90-day all-cause mortality rates were also similar, 11.9% versus 8.8%, respectively (*p* = 0.086). Compared with patients with influenza, patients with RSV infections were similar in age but more frequently had underlying chronic lung diseases and major systemic comorbidities [27].

All these observations are important because, while pediatricians are keenly aware that RSV can cause serious illness in their patients, most clinicians are less aware of the impact of RSV on adults, especially frail older patients [17]. Natural RSV infection does not confer long-lasting immunity against subsequent infection, and there are no approved treatments or preventive vaccines against RSV infection that effectively protect vulnerable adult populations from hospitalization and mortality [22,34]. Prevention remains the most effective strategy to control RSV infection, including visiting restrictions during the RSV outbreak and general hygienic practices, particularly among high-risk adult populations. In addition, it is essential to quickly carry out diagnostic confirmation tests in all patients suspected of having RSV infection in order to offer the most appropriate therapy using the available means [22,34]. These results highlight the unmet need for the development of effective and safe preventive and treatment options, including antiviral agents and vaccines for the management of RSV infections, particularly among high-risk and older adults.

This study has strengths and limitations that should be mentioned. Its main strength lies in the use of MBDS records that offer a very large sample size that includes practically all admissions to both public and private acute hospitals in Spain, thus increasing the power of the statistical analyses, even for low-prevalence diseases [35]. However, one of the main limitations when interpreting the MBDS data is that they rely on the quality of the discharge report and the clinical history and on the codification process variables [36]. Confirmatory laboratory tests are not always performed when RSV infection is suspected during hospitalization, and even if testing is performed, the differences in the RSV coding behavior of physicians, which may vary during and out of RSV season for many reasons, may lead to information bias. Although RSV is a common pathogen of acute lower respiratory infections and can cause severe illness in adults, it normally goes unrecognized in individuals with milder symptoms, so the real impact of RSV infections may be underestimated in this population [37].

## 5. Conclusions

In summary, our results highlight the substantial burden of RSV respiratory infections in the adult population in Spain, whose impact is not fully realized by most internists. Adult patients admitted for ALRTIs, particularly frail and high-risk patients, should undergo laboratory testing for both influenza and RSV viruses. Until a vaccine is available, early protective measures should be applied to address this problem, including general hygienic practices, such as visiting restrictions during the RSV outbreak period and effective handwashing. When an RSV vaccine becomes available, the most effective vaccination strategies for the population should be defined. According to the results of a recent study, vaccination of children under 5 years of age may be the most effective prevention strategy, not only for children but also for older adults, as this will reduce transmission, and the indirect protection offered by this approach can prevent even more infections than individual protection in adults over 50 years of age [38].

## Figures and Tables

**Table 1:** Definition of respiratory system diseases used according to the ICD-9-CM and ICD-10-CM codes.

ICD-9-CM Codes (Cases Collected from 2012 to 2015)	
079.6	Respiratory syncytial virus infection
466.0	Acute bronchitis
466.1	Acute bronchiolitis
466.11	Acute bronchiolitis due to respiratory syncytial virus
480.1	Pneumonia due to respiratory syncytial virus
487.0	Influenza with pneumonia
487.1	Influenza with other respiratory manifestations
490	Bronchitis, not specified as acute or chronic
493.9	Asthma unspecified
**ICD-10-CM Codes (Cases Collected from 2016 to 2020)**	
B97.4	Respiratory syncytial virus as the cause of diseases classified elsewhere
J12.1	Respiratory syncytial virus pneumonia
J20.5	Acute bronchitis due to respiratory syncytial virus
J20.9	Acute bronchitis, unspecified
J21.0	Acute bronchiolitis due to respiratory syncytial virus
J21.9	Acute bronchiolitis, unspecified
J11.0	Influenza due to unidentified influenza virus with pneumonia
J11.1	Influenza due to unidentified influenza virus with other respiratory manifestations
J40	Bronchitis, not specified as acute or chronic
J45.9	Other and unspecified asthma

**Table 2:** The hospitalization rate in the general population in Spain by diagnostic group and year between 2012 and 2020.

	Nonspecified Infection		RSV-Related Infection		Influenza-Related Infection	
Year	N	Annual Rate ^a^	N	Annual Rate	N	Annual Rate
2012	129,628	28.89	13,647	2.22	2527	0.54
2013	133,969	29.66	11,262	1.94	3146	0.66
2014	140,318	30.93	13,018	2.33	4739	1.03
2015	149,398	32.86	14,659	2.65	9045	2.00
2016	143,298	31.38	15,089	2.80	7207	1.56
2017	159,644	34.74	16,973	3.29	7064	1.55
2018	172,387	37.22	18,726	3.76	8697	1.89
2019	168,735	35.98	22,180	4.59	2369	0.50
2020	136,785	28.88	12,240	2.58	1494	0.32
Total	1,334,162	31.74	137,794	3.28	46,288	1.10
*p*-value	<0.001		<0.001		<0.001	

^a^ Mean annual hospitalization rates expressed per 100,000 population. HR—hospitalization rate; N—number of patients; RSV—respiratory syncytial virus.

**Table 3:** Hospitalization rate in the general population in Spain by diagnostic group and age group between 2012 and 2020.

	Nonspecified Infection		RSV-Related Infection		Influenza-Related Infection	
Age Group	N (%)	Annual Rate ^a^	N (%)	Annual Rate ^a^	N (%)	Annual Rate ^a^
<1 yr	61,053 (4.6)	168.15	95,647 (69.4)	263.42	2593 (5.6)	7.14
		(167.77–168.53)		(262.97–263.87)		(7.05–7.23)
1 yr	23,334 (1.7)	61.06	12,744 (9.2)	33.35	1253 (2.7)	3.28
		(60.06–61.3)		(33.17–33.53)		(3.22–3.34)
2–4 yr	36,447 (2.7)	29.76	7337 (5.3)	5.99	2047 (4.4)	1.67
		(29.66–29.86)		(5.95–6.03)		(1.65–1.69)
5–14 yr	40,500 (3.0)	9.38	1348 (1.0)	0.31	1851 (4.0)	0.43
		(9.35–9.41)		(0.30–0.32)		(0.42–0.44)
15–19 yr	18,509 (1.4)	9.18	133 (0.1)	0.07	348 (0.8)	0.17
		(9.14–9.22)		(0.07–0.07)		(0.16–0.18)
20–29 yr	57,204 (4.3)	12.79	244 (0.2)	0.05	1034 (2.2)	0.23
		(12.76–12.82)		(0.05–0.05)		(0.23–0.23)
30–39 yr	94,060 (7.1)	15.22	433 (0.3)	0.07	2016 (4.4)	0.30
		(15.19–15.25)		(0.07–0.07)		(0.33–0.33)
40–49 yr	96,217 (7.2)	13.95	857 (0.6)	0.12	2892 (6.2)	0.42
		(13.92–13.98)		(0.12–0.12)		(0.42–0.42)
50–59 yr	115,098 (8.6)	19.45	1739 (1.3)	0.29	4337 (9.4)	0.73
		(19.41–19.49)		(0.29–0.29)		(0.72–0.74)
60–69 yr ^b^	155,522 (11.7)	34.46	3023 (2.2)	0.67	6318 (13.6)	1.40
		(34.41–34.51)		(0.66–0.68)		(1.39–1.41)
70–79 yr	241,101 (18.1)	73.67	4830 (3.5)	1.48	8535 (18.4)	2.61
		(73.58–73.76)		(1.47–1.49)		(2.59–2.63)
80–89 yr	307,287 (23.0)	149.82	7065 (5.1)	3.44	10,180 (22.0)	4.96
		(149.67–149.97)		(3.41–3.47)		(4.93–4.99)
≥90 yr	87,830 (6.6)	204.03	2394 (1.7)	5.56	2884 (6.2)	6.70
		(203.65–204.41)		(5.49–5.63)		(6.62–6.78)
Total	1,334,162 (100.0)	31.74	137,794 (100.0)	3.28	46,288 (100.0)	1.10
		(31.72–31.76)		(3.27–3.29)		(1.1–1.1)
*p*-value		<0.001		<0.001		<0.001

^a^ Mean annual hospitalization rates expressed per 100,000 population (95% CI). ^b^ The mean annual hospitalization rates (per 100,000 population) among patients aged 60 years or older were estimated at: 77.11 (95% CI 77.06–77.16) for patients in the “nonspecified lower respiratory tract infections” diagnostic group, 1.69 (95% CI 1.68–1.70) for patients in the “RSV-related infection” diagnostic group and 2.72 (95% CI 2.71–2.73) for patients in the “influenza-related infections” diagnostic group. N (%)—number of patients (% of patients with respect to the total number of patients in the group); RSV—respiratory syncytial virus; yr—years.

**Table 4:** In-hospital case fatality rate in the general population in Spain by diagnostic group and year between 2012 and 2020.

	Nonspecified Infection		RSV-Related Infection		Influenza-Related Infection	
Year	N	CFR ^a^	N	CFR	N	CFR
2012	4604	3.55	34	0.25	93	3.68
2013	4599	3.43	33	0.29	102	3.24
2014	4904	3.49	57	0.44	222	4.68
2015	5723	3.83	69	0.47	472	5.22
2016	5383	3.76	105	0.70	249	3.45
2017	6257	3.92	201	1.18	429	6.07
2018	6682	3.88	299	1.60	463	5.32
2019	6397	3.79	452	2.04	144	6.08
2020	6759	4.94	401	3.28	80	5.35
Total	51,308	3.85	1651	1.20	2254	4.87
*p*-value	<0.001		<0.001		<0.001	

^a^ In-hospital case-fatality rate expressed as a percentage of deaths per inpatient population (95% CI). CFR—in-hospital case fatality rate; N—number of patients; RSV—respiratory syncytial virus.

**Table 5:** The in-hospital case fatality rate in the general population in Spain by age group and diagnostic group between 2012 and 2020.

	Nonspecified Infection		RSV-Related Infection		Influenza-Related Infection	
Age Group	N (%)	Annual Rate ^a^	N (%)	Annual Rate ^a^	N (%)	Annual Rate ^a^
<1 yr	53 (0.1)	0.09	84 (5.1)	0.09	4 (0.2)	0.15
		(0.07–0.11)		(0.07–0.11)		(0–0.3)
1 yr	17 (0.0)	0.07	19 (1.2)	0.15	3 (0.1)	0.24
		(0.04–0.1)		(0.08–0.22)		(0.03–0.51)
2–4 yr	15 (0.0)	0.04	17 (1.0)	0.23	2 (0.1)	0.10
		(0.02–0.06)		(0.12–0.34)		(0.04–0.24)
5–14 yr	49 (0.1)	0.12	25 (1.5)	1.85	21 (0.9)	1.13
		(0.09–0.15)		(1.13–2.57)		(0.65–1.61)
15–19 yr	43 (0.1)	0.23	3 (0.2)	2.26	4 (0.2)	1.15
		(0.16–0.3)		(0.27–4.79)		(0.03–2.27)
20–29 yr	157 (0.3)	0.27	9 (0.5)	3.69	18 (0.8)	1.74
		(0.23–0.31)		(1.32–6.06)		(0.94–2.54)
30–39 yr	374 (0.7)	0.40	12 (0.7)	2.77	23 (1.0)	1.14
		(0.36–0.44)		(1.22–4.32)		(0.68–1.6)
40–49 yr	1054 (2.1)	1.10	36 (2.2)	4.20	71 (3.1)	2.46
		(1.03–1.17)		(2.86–5.54)		(1.9–3.02)
50–59 yr	2424 (4.7)	2.11	76 (4.6)	4.37	178 (7.9)	4.10
		(2.03–2.19)		(3.41–5.33)		(3.51–4.69)
60–69 yr ^b^	4701 (9.2)	3.02	179 (10.8)	5.92	284 (12.6)	4.50
		(2.93–3.11)		(5.08–6.76)		(3.99–5.01)
70–79 yr	9988 (19.5)	4.14	305 (18.5)	6.31	451 (20.0)	5.28
		(4.06–4.22)		(5.62–7)		(4.81–5.75)
80–89 yr	21,994 (42.9)	7.16	600 (36.3)	8.49	791 (35.1)	7.77
		(7.07–7.25)		(7.84–9.14)		(7.25–8.29)
≥90 yr	10,439 (20.3)	11.89	286 (17.3)	11.95	404 (17.9)	14.01
		(11.68–12.1)		(10.65–13.25)		(12.74–15.28)
Total	51,308 (100.0)	3.85	1651 (100.0)	1.20	2254 (100.0)	4.87
		(3.82–3.88)		(1.14–1.26)		(4.67–5.07)
*p*-value		<0.001		<0.001		<0.001

^a^ In-hospital case-fatality rate expressed as a percentage of deaths per inpatient population (95% CI). ^b^ The in-hospital case-fatality rates among patients aged 60 years or older were estimated at: 5.95% (95% CI 5.94–5.96) for patients in the “nonspecified lower respiratory tract infections” diagnostic group, 7.91% (95% CI 7.89–7.93) for patients in the “RSV-related infection” diagnostic group and 6.91% (95% CI 6.89–6.93) for patients in the “influenza-related infection” diagnostic group. N (%)—number of patients (% of patients with respect to the total number of patients in the group); RSV—respiratory syncytial virus; yr—years.

## Data Availability

The datasets analyzed in the current study are publicly available in the Hospital Discharge Records in the Spanish National Health System (CMBD) repository at: https://www.mscbs.gob.es/en/estadEstudios/estadisticas/cmbdhome.htm (accessed on 5 August 2022). The information contained in this repository can be accessed without the need for any administrative permissions.
